# “Esketamine” in Borderline Personality Disorder: focud on suicide ideation

**DOI:** 10.1192/j.eurpsy.2024.1356

**Published:** 2024-08-27

**Authors:** M. Olivola, F. Mazzoni, V. Arienti, S. C. Civardi, G. Carnevale Miacca, P. Leali, F. Santilli, A. Guffanti, N. Brondino

**Affiliations:** Department of Brain and behavioral science, University of Pavia, Pavia, Italy

## Abstract

**Introduction:**

Borderline personality disorder is often associated with comorbid conditions such as eating disorders, mood disorders, and substance use disorders. The prevalence of BPD and major depressive disorder (MDD) are about 5.9% and 8%, respectively, but up to 80% of patients with BPD experience one or more episodes of MDD in their lifetime. BPD is associated with suicidal behaviors and self-harm, thei are also fifty times more likely than the general population to attempt or die by suicide, Up to 10% of BPD patients will die by suicide

**Objectives:**

Our aim is to verify if Esketamine could be effectiveness in treating patterns of behavior that have proven to be socially disruptive like self harm, suicidal attempts in patients with BPD. Suicidal ideation is a major risk factor for suicide in patients with TRD and BPD. The interval between the onset of suicidal ideation and suicide attempt is often very short, highlighting the need for urgent intervention and the development of new rapid-onset antidepressant therapies.

**Methods:**

We recruited 25 adult subjects referred to the outpatient clinics of Pavia suffering from TRD with current Moderate-Severe Depressive Episode (scoring ≥ 22 on the MADRS). Of them 9/25 patients has a BPD. Study duration was 8 weeks.The following evaluation scales were administered before the first drug administration (T0) and repeated after one week (T1), four weeks (T2) and eight weeks (T3) of treatment: Montgomery Asberg Depression Rating Scale (MADRS), Columbia-Suicide Severity Rating Scale (CSSRS), and The Zanarini Rating Scale for BPD subgroup patients. We also collected sociodemographic and clinical information. Dosages and frequency of esketamine administration during the study period, adverse events and reasons for discontinuation were also recorded.

**Results:**

A significant reduction of depressive symptoms was found at T1 and T2 compared to T0. Suicidal ideation disappeared as early as T1 and was maintained at T2, expecially in the BPD group. In the subgroup with borderline disorder we saw more improvement in impulsive (Self-mutilation and/or suicidal efforts; two other forms of impulsivity) and affective categories ( Inappropriate anger / frequent angry acts; chronic feelings of emptiness; mood instability) in Zanarini Rating Scale.

**Conclusions:**

Our findings support the safety and tolerability of esketamine in TRD and BPD comorbidity sample. It is noteworthy that esketamine has an action on various pathways that are considered defective in borderline patients. Glutamate plays a key role in personality traits such as impulsivity, aggression, and suicidal behavior. Treatment with esketamine could reduce the number of suicide attempts and help reduce the self-harm of BPD.

**Disclosure of Interest:**

None Declared

